# Bibliographical

**Published:** 1873-07

**Authors:** 


### BIBLIOGRAPHICAL.

*A Treatise on the Principles and Practice of Medicine*—Designed for the Use of Practitioners and Students of Medicine. By AUSTIN FLINT, M.D., Professor of the Principles and Practice of Medicine, and of Clinical Medicine, in the Bellevue Hospital Medical College. Fourth Edition, Carefully Revised, and Published by Henry C. Lea, Philadelphia.

It is of course useless to say anything in regard to the merits of a work with which every intelligent practitioner of medicine is familiar, and we can not do better than, in the language of the author, to say: "Since the publication of the third edition of this work, in October, 1868, the advances made in many departments of Practical Medicine have seemed to call for a thorough revision. In the performance of this duty the author has sought to introduce the results of his continued clinical studies, together with the latest contributions to medical literature, both in Europe and America, without changing the character of the volume as a condensed text-book and work of reference. This has required the rewriting of some portions, as well as numerous additions throughout, but more especially in the sections devoted to the diseases of the nervous system, causing an increase in the text of about seventy pages."

*Mineral Springs of North America—How to Reach and How to Use Them.* By J. J. MOORMAN, M.D., Physician to the White Sulphur Springs; Professor of Medical Jurisprudence and Hygiene in Washington University, Baltimore; Member of the Medico-Chirurgical Society of Maryland, and of the Baltimore Medical Association, etc. J. B. Lippincott & Co., Publishers, Philadelphia.

This is an enlargement of the writings of Dr. Moorman, in book form, upon the history and medicinal application of mineral waters, commenced in 1839, as a "Directory" for the use of the water of the Greenbrier White Sulphur Springs, in Virginia, and enlarged into a volume published in 1846, under the title of "Virginia Springs," new editions of which were issued in 1855 and 1857. A new edition of a more comprehensive character, embracing not only the Virginia Springs, but the springs of the Southern and Western States, was published in 1859, and again in 1867 a more comprehensive volume was issued entitled "Mineral Springs of the United States and Canada," and now, as we learn from the author, important mineral waters in the

United States and the continent having been brought into practical use, with the obviously growing importance in the public mind of mineral waters generally as remedial agents, he has still further increased the scope of the volume and published the work before us under the title of "Mineral Springs of North America." The long experience of the author in observing the effects of and prescribing mineral waters, and the eminently practical character of his book, enable us to commend it to all those who feel interested in the rapidly-growing importance of mineral waters as therapeutic agents.

*The Mineral Springs of the United States and Canada, with Analyses and Notes on the Prominent Spas of Europe, and a List of Seaside Resorts.* By GEO. WALTON, M.D., Lecturer on Materia Medica in the Miami Medical College, Cincinnati, and Committee of the Medical Association of Ohio, on "Therapeutics of Mineral Springs." D. Appleton & Co., Publishers, 549 and 551 Broadway, N.Y.

While we think the author of this work has claimed too much for himself in ignoring the labors of others, and especially in depreciating the knowledge already acquired in regard to the action of mineral waters, we concede to it an attempt at a rather more scientific discusssion of the therapeutic action of mineral waters than can, perhaps, be found elsewhere, and, as a valuable addition to the literature of this subject, we would commend it.

